# Social Determinants of Health and Tobacco Use in Thirteen Low and Middle Income Countries: Evidence from Global Adult Tobacco Survey

**DOI:** 10.1371/journal.pone.0033466

**Published:** 2012-03-16

**Authors:** Krishna M. Palipudi, Prakash C. Gupta, Dhirendra N. Sinha, Linda J. Andes, Samira Asma, Tim McAfee, on behalf of the GATS Collaborative Group

**Affiliations:** 1 Centers for Disease Control and Prevention, Atlanta, Georgia, United States of America; 2 Healis Sekhsaria Institute for Public Health, Mumbai, India; 3 South East Asia Regional Office, World Health Organization, New Delhi, India; Fundación para la Prevención y el Control de las Enfermedades Crónicas No Transmisibles en América Latina (FunPRECAL), Argentina

## Abstract

**Background:**

Tobacco use has been identified as the single biggest cause of inequality in morbidity. The objective of this study is to examine the role of social determinants on current tobacco use in thirteen low-and-middle income countries.

**Methodology/Principal Findings:**

We used nationally representative data from the Global Adult Tobacco Survey (GATS) conducted during 2008–2010 in 13 low-and-middle income countries: Bangladesh, China, Egypt, India, Mexico, Philippines, Poland, Russian Federation, Thailand, Turkey, Ukraine, Uruguay, and Viet Nam. These surveys provided information on 209,027 respondent's aged 15 years and above and the country datasets were analyzed individually for estimating current tobacco use across various socio-demographic factors (gender, age, place of residence, education, wealth index, and knowledge on harmful effects of smoking). Multiple logistic regression analysis was used to predict the impact of these determinants on current tobacco use status. Current tobacco use was defined as current smoking or use of smokeless tobacco, either daily or occasionally. Former smokers were excluded from the analysis. Adjusted odds ratios for current tobacco use after controlling other cofactors, was significantly higher for males across all countries and for urban areas in eight of the 13 countries. For educational level, the trend was significant in Bangladesh, Egypt, India, Philippines and Thailand demonstrating decreasing prevalence of tobacco use with increasing levels of education. For wealth index, the trend of decreasing prevalence of tobacco use with increasing wealth was significant for Bangladesh, India, Philippines, Thailand, Turkey, Ukraine, Uruguay and Viet Nam. The trend of decreasing prevalence with increasing levels of knowledge on harmful effects of smoking was significant in China, India, Philippines, Poland, Russian Federation, Thailand, Ukraine and Viet Nam.

**Conclusions/Significance:**

These findings demonstrate a significant but varied role of social determinants on current tobacco use within and across countries.

## Introduction

Socioeconomic inequality and its impact on health is a global public health concern [1]. Smoking has been identified as the single biggest cause of inequality in morbidity and mortality between rich and poor people in many countries [2]. Studies from Western countries have reported an association between social and economic determinants and smoking to the detriment of those in the disadvantaged groups [3]. Several independent studies at international level [4], national level [5] and sub national [6] level from developing countries have shown association of tobacco use with social and economic determinants such as age, education, gender, occupation, ethnicity and place of residence.

National data on prevalence of tobacco use (with some limitations on age groups and gender representation) have been available from Demographic Health Surveys in Bangladesh [7], Egypt [8], India [9], Philippines [10], Turkey [11], Ukraine [12] and Vietnam [13]. These data indicate tobacco use is higher among males, and among disadvantaged sections of society characterized by people living in rural areas and with low education, and lower socioeconomic status. However, the information on tobacco use was only peripheral rather than an objective of these surveys and therefore, the questions on tobacco use were not standardized across countries or even within different surveys in a country.

Global Adult Tobacco Survey (GATS) is a component of the Global Tobacco Surveillance System (GTSS) which includes: the Global Youth Tobacco Survey (GYTS); the Global School Personnel Survey (GSPS); and the Global Health Professions Student Survey (GHPSS). The objectives of GATS in its first phase of implementation was to monitor tobacco use and tobacco control indicators in low and middle income countries bearing the highest burden based on number of adults smokers. The first phase of GATS was implemented in 14 countries during 2008–2010: Bangladesh, Brazil, China, Egypt, India, Mexico, Philippines, Poland, Russian Federation, Thailand, Turkey, Ukraine, Uruguay, and Viet Nam.

GATS is a global standard for systematically monitoring adult tobacco use and tracking key tobacco control indicators. GATS is a nationally representative household survey of adults aged 15 years and older, using a consistent and standard protocol which enables unprecedented cross-country comparisons and change over time for countries that repeat the survey. This paper examines the influence of various socio-demographic variables on current tobacco use within a country and across countries using GATS data.

## Materials and Methods

### Study Area and Source of Data

GATS data from 13 low-and-middle income countries (Bangladesh, China, Egypt, India, Mexico, Philippines, Poland, Russian Federation, Thailand, Turkey, Ukraine, Uruguay, and Viet Nam) conducted during 2008–2010 were used for analyses. GATS data from Brazil was not included in this paper as the information on important predictor variables collected in Brazil (education, wealth index) was not comparable to other GATS countries. These surveys provided information on 209,027 respondent's aged 15 years and above and the country datasets were analyzed individually for estimating overall current tobacco use as well as by various socio-demographic factors.

GATS used a multi-stage geographically clustered sample design to produce nationally representative data. For each participating country, a standard protocol with respect to questionnaire, sample design, data collection and management procedures was used. Survey information was collected using handheld devices. Additional details of individual country survey methodologies are available in country reports [Bibr pone.0033466-World1] [Bibr pone.0033466-GATS1] [Bibr pone.0033466-The1] [Bibr pone.0033466-International2] [Bibr pone.0033466-Ministry2] [Bibr pone.0033466-GATS2] [Bibr pone.0033466-Ministry3] [Bibr pone.0033466-GATS3] [Bibr pone.0033466-World2] [Bibr pone.0033466-The2] [Bibr pone.0033466-GATS4] [Bibr pone.0033466-GATS5] [14 to 26]. The list of GATS collaborating group in the 14 countries and other partner organizations is provided in List S1.

### Variables Included in the Analyses

Current tobacco use is the dependent variable used in this analysis and was defined as current smoking or use of any smokeless tobacco product, either daily or occasionally [27] using the following questions: 1) ‘Do you currently smoke tobacco on a daily basis, less than daily, or not at all’ and 2) ‘Do you currently use smokeless tobacco on a daily basis, less than daily, or not at all’. Out of all 13 countries, only Turkey did not ask the questions on smokeless tobacco. Former tobacco users were defined as the number of ever tobacco smokers or smokeless tobacco users who currently do not smoke or use any form of tobacco. Never tobacco users were defined as adults who reported that they neither smoked nor used smokeless tobacco in their life time.

Relevant independent variables included in the analyses were gender (male/female), age, place of residence (urban/rural), knowledge on harmful effects of smoking (three categories), educational level (four categories), and wealth index (five categories). The level of knowledge on harmful effects of smoking was measured using three core questions in each country: ‘based on what you know or believe does smoking tobacco cause the following: stroke (blood clots in the brain that may cause paralysis), heart attack and lung cancer’. Respondents who answered all three questions correctly were classified as having ‘good knowledge’, those who answered any two questions correctly as having ‘some knowledge’ and rest were classified as having ‘little knowledge’. Educational level was grouped into five categories: no formal schooling, less than primary, primary complete, less than secondary, and secondary school complete and above (includes high school, college/university, and post graduate and above education) across all countries. Wealth index, a proxy measure for respondent socioeconomic status, was constructed using principal component analysis with information on household ownership of assets [28]. The asset information included whether households possessed such items as electricity, flush toilet, fixed telephone, cell telephone, television, radio, refrigerator, car, moped/scooter/motorcycle, washing machine, etc. The sample was divided into quintiles from one (lowest) to five (highest) for each country. A single wealth index was developed for the whole respondent sample. Thus, at a national level, for each country, 20 percent of the sample respondents are in each wealth quintile although indexes it is not necessarily true at population level.

### Statistical Analysis

The data were appropriately weighted to ensure the true representation of the population of the country; SPSS® version 18.0 for complex samples was used to analyze the data. Statistical analysis included multiple logistic regression accounted for complex survey design for predicting the social determinants of tobacco use. The dependent variable used for this analysis was tobacco use (tobacco user-1; never tobacco user-0). Former tobacco users were removed from the logistic regression analysis due to the fact that current tobacco use may not directly influence from current socioeconomic and demographic status. All the independent variables were categorical. Overall trend for each variable was assessed by assuming the categories of independent variables as continuous variables in the logistic regression, except for age variable where we used age in single years for obtaining the trend.

## Results

Table 1 shows the sample characteristics for 13 countries. Since sample design in each country was stratified by gender and place of residence (urban/rural), the distributions for these two variables reflect the population distribution. The age distribution showed a steep pyramidal structure for five countries (Bangladesh, Egypt, India, Mexico and Philippines). The education distribution showed a step gradient for Bangladesh and India. In four countries, over one third of the sample has no formal or less than primary education (Bangladesh, Egypt, India and Thailand). The percent distribution of adult population by wealth quintiles, based on the household assets included in the survey shows more or less an even distribution across many countries except few exemptions (e.g. India, Mexico, Russia, Ukraine, and Uruguay) where a varied socioeconomic status of the population was observed. For example, almost 28% of respondents in India were classified as having lowest wealth index whereas only 11.2% fall under lowest wealth quintile in Mexico. The level of knowledge on harmful effects of smoking varied a great deal across countries. Interestingly, the highest level ‘good knowledge’ was reported most in Egypt (88%) and least in China (less than 23%).

**Table 1:** Distribution of adults aged 15 years and above by socio-demographic characteristics in 13 low-and-middle income countries, Global Adult Tobacco Survey, 2008–2010.

Socio-demographic characteristics	Bangladesh	China	Egypt	India	Mexico	Philippines	Poland	Russian Federation	Thailand	Turkey	Ukraine	Uruguay	Viet Nam
	(n = 9,629)	(n = 13,354)	(n = 20,924)	(n = 69,296)	(n = 13,617)	(n = 9,701)	(n = 7,840)	(n = 11,406)	(n = 20,566)	(n = 9,030)	(n = 8,158)	(n = 5,581)	(n = 9,925)
**Gender**													
Male	49.7	50.9	51.0	51.7	47.7	49.9	47.7	45.3	48.6	49.1	45.4	47.4	48.6
Female	50.3	49.1	49.0	48.3	52.3	50.1	52.3	54.7	51.4	50.9	54.6	52.6	51.4
**Age**													
15–24	29.5	21.5	33.0	29.5	27.7	29.6	17.3	17.8	20.0	22.5	18.4	20.2	25.9
25–34	23.5	15.4	22.4	22.7	23.3	21.4	18.9	18.0	18.2	23.5	18.0	18.6	19.7
35–44	19.6	24.1	17.6	19.3	19.1	20.6	15.8	16.5	23.2	19.2	16.3	16.8	22.2
45–54	12.8	15.7	13.5	12.9	13.2	13.0	17.5	19.0	16.8	15.5	16.0	14.8	15.0
55–64	8.0	13.5	8.0	8.8	8.6	8.9	14.6	12.8	12.1	10.1	14.0	12.4	8.4
65+	6.6	9.8	5.5	6.8	8.0	6.5	15.9	15.9	9.7	9.2	17.3	17.2	8.8
**Place of residence**													
Urban	26.2	46.1	45.3	29.2	77.8	49.8	62.0	74.5	31.1	69.7	68.0	92.7	30.7
Rural	73.8	53.9	54.7	70.8	22.2	50.2	38.0	25.5	68.9	30.3	32.0	7.3	69.3
**Education**													
No formal education/Less than primary	51.3	15.9	41.0	43.2	18.3	21.5	1.4	0.3	33.7	17.1	0.8	10.6	21.3
Completed primary/Less than secondary	33.8	18.4	6.3	28.9	23.6	17.2	14.3	3.8	21.0	48.8	10.3	36.5	24.3
Completed secondary/high school	11.5	53.4	41.5	19.6	49.7	39.1	69.1	65.2	37.3	25.2	66.5	45.0	48.3
Completed college/university or above	3.3	12.2	11.1	8.3	8.4	22.3	15.2	30.7	8.0	8.9	22.3	7.9	6.1
**Wealth Index**													
Lowest	19.2	15.7	21.9	28.0	11.2	18.1	16.3	14.8	19.2	14.5	14.2	11.9	20.6
Low	21.6	18.1	19.7	18.4	15.4	18.1	19.3	16.4	21.2	20.4	15.4	16.9	24.0
Medium	23.4	20.4	17.6	24.1	19.3	20.7	18.7	21.9	20.6	21.1	20.6	19.4	21.9
High	20.8	21.4	23.0	15.5	24.1	20.4	23.5	12.3	23.1	23.8	22.9	22.0	17.6
Highest	15.0	24.5	17.8	14.0	30.0	22.7	22.2	34.5	15.9	20.3	26.9	29.7	16.0
**Knowledge**													
Little knowledge	12.9	57.5	5.1	33.1	16.5	18.4	18.7	25.9	15.5	6.0	17.7	7.0	22.9
Some knowledge	8.4	20.4	6.9	22.5	27.3	12.1	21.4	10.4	14.1	12.7	9.1	18.0	21.5
Good knowledge	78.7	22.1	88.0	44.5	56.2	69.6	59.8	63.7	70.4	81.2	73.2	75.0	55.5
**Tobacco Use**													
Current tobacco user	43.3	28.1	19.7	34.6	16.0	29.5	30.5	39.3	27.2	31.2	28.9	25.0	25.0
Never tobacco user	53.0	66.5	75.8	62.4	69.2	59.5	47.5	46.7	60.6	52.8	55.9	51.1	64.9
Former tobacco user	3.7	5.4	4.4	3.0	14.7	11.0	22.0	13.9	12.2	15.9	15.2	24.0	10.1

**Note:** n - sample size.

Table 2 shows the prevalence of tobacco use by various socio-demographic factors. The prevalence of current tobacco use varied from 16% in Mexico to 43.3% in Bangladesh. Former users varied much more, from 3% in India to 24% in Uruguay. Among males, the prevalence varied from 25% in Mexico to 60.6% in the Russian Federation. Among females, the variation was much higher from 0.6% in Egypt to 28.7% in Bangladesh. Prevalence varied considerably by gender, the level of education and wealth index and by the level of knowledge on effects of smoking.

**Table 2:** Prevalence of current tobacco use among adults aged 15 years and above by socio-demographic characteristics in 13 low-and-middle income countries, Global Adult Tobacco Survey, 2008–2010.

Socio-demographic characteristics	Bangladesh	China	Egypt	India	Mexico	Philippines	Poland	Russian Federation	Thailand	Turkey	Ukraine	Uruguay	Viet Nam
**Overall**	43.3	28.1	19.7	34.6	16.0	29.5	30.5	39.3	27.2	31.2	28.9	25.0	25.0
**Gender**													
Male	58.0	52.9	38.1	47.9	25.0	49.2	37.3	60.6	46.4	47.9	50.1	30.7	47.6
Female	28.7	2.4	0.6	20.3	7.9	10.0	24.4	21.7	9.1	15.2	11.3	19.8	3.6
**Age**													
15–24	16.9	17.9	11.3	18.4	17.0	21.2	24.7	43.1	19.8	25.3	30.5	24.7	13.3
25–34	36.3	28.7	22.3	33.2	17.9	33.5	34.1	51.1	25.6	40.4	42.0	34.7	26.0
35–44	55.0	32.4	24.5	42.2	16.1	32.3	36.6	48.2	27.8	39.4	38.0	25.7	31.7
45–54	67.6	36.0	26.7	45.5	17.7	33.8	43.1	41.8	26.5	32.6	32.4	32.4	31.2
55–64	70.7	30.9	24.3	49.4	12.9	33.1	31.6	32.8	31.7	24.7	20.8	24.1	30.1
65+	70.8	22.7	20.5	47.8	8.1	32.0	11.8	14.9	39.8	10.3	8.5	8.1	24.5
**Place of residence**													
Urban	38.1	26.1	19.8	25.3	17.5	25.3	32.0	40.5	22.9	33.0	30.5	25.1	23.6
Rural	45.1	29.9	19.7	38.4	11.0	33.7	28.0	36.0	29.2	27.2	25.6	23.4	25.6
**Education**													
No formal education/Less than primary	58.1	20.9	22.7	44.6	11.5	45.1	11.6	23.9	34.3	15.0	15.7	24.4	28.4
Completed primary/Less than secondary	30.3	28.5	21.6	32.7	15.4	33.6	23.9	17.6	29.5	31.4	15.5	27.1	28.2
Completed secondary/high school	19.3	31.5	17.5	21.7	17.6	26.6	33.6	42.0	23.0	42.0	32.5	24.1	22.4
Completed college/university or above	29.7	22.6	16.2	18.3	18.5	16.4	24.8	36.5	11.7	31.8	24.6	20.6	20.5
**Wealth Index**													
Lowest	55.5	29.0	20.7	47.6	10.9	40.4	27.8	36.8	37.0	26.4	24.8	37.7	30.3
Low	47.4	30.6	21.8	38.4	12.1	35.6	32.2	35.3	31.8	33.6	27.3	25.7	27.7
Middle	43.5	28.3	23.1	32.4	15.4	31.7	32.8	39.8	27.7	32.8	31.4	27.1	24.1
High	38.6	28.7	19.4	25.5	17.8	26.9	30.4	35.2	23.3	31.9	28.5	22.2	21.9
Highest	28.1	25.2	13.3	17.2	19.0	16.4	29.4	43.6	14.5	30.0	30.5	20.1	18.6
**Knowledge on effects of smoking**													
Little knowledge	50.6	30.2	22.3	41.7	13.4	42.9	46.4	56.7	32.7	29.2	42.1	32.6	34.0
Some knowledge	38.4	25.4	21.1	31.9	18.3	30.7	31.0	44.2	26.9	31.7	34.8	22.8	23.2
Good knowledge	42.7	25.0	19.5	30.5	15.8	25.8	25.4	31.5	26.1	31.3	25.0	24.8	21.9
**Number of tobacco users (in millions)**	41.3	300.8	9.8	274.9	11.0	18.1	9.9	44.2	14.3	16.0	11.6	0.6	16.1

**Figure 1:**
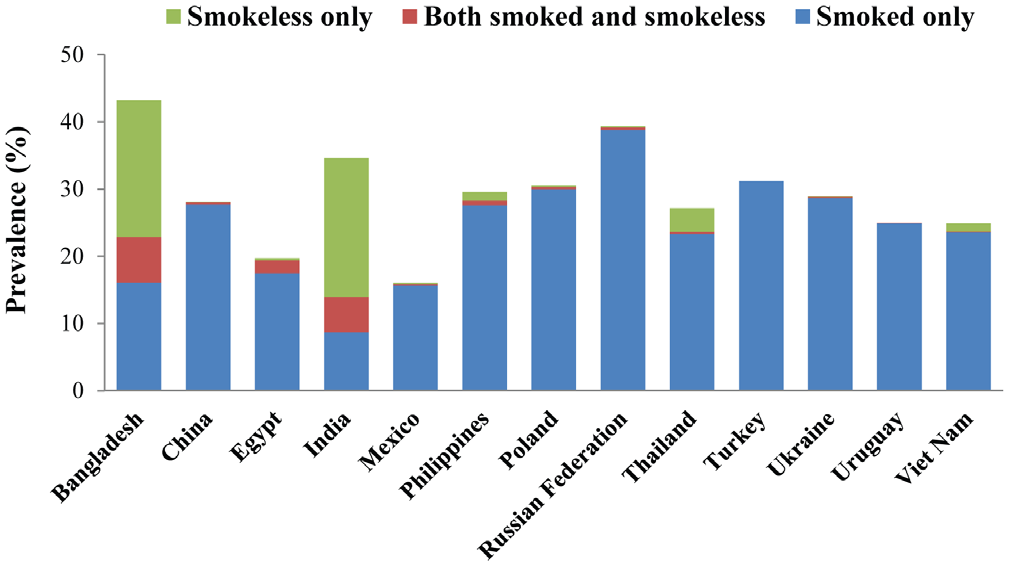
Type of current tobacco use among adults aged 15 years and above in 13 low-and-middle income countries, Global Adult Tobacco Survey, 2008–2010.

Tobacco use included smoking, smokeless tobacco use and dual use (using both smoked and smokeless). Figure 1 shows the type of tobacco use. It is clear that in Bangladesh and India, smokeless tobacco use constitutes a major part of overall tobacco use. In Thailand, Philippines and Viet Nam smokeless tobacco use also makes some contribution to overall tobacco use. In addition, in the countries where smokeless tobacco use prevalence is high along with smoking, dual use (use of both smoking and smokeless tobacco products) also contributes to a noticeable proportion and somewhat more likely in those countries (e.g. Bangladesh (8.7%) and India (5.3%)).

Both Table 3 and Table 4 show the odds ratios for current tobacco use versus no tobacco use using a multiple logistic regression model incorporating all variables in the table. Odds ratios were significantly higher for males in all countries with great variation across countries (from 2.1 in Uruguay to 161.9 in Egypt). As shown in Table 2, the prevalence of current smokers is quite lower among women compared with men in most countries and also sex of the respondent was a very strong determinant of smoking status (Table 3–4). Compared to the lowest age group (15–24 years), odds ratios were significantly higher in almost all age groups in almost all countries with very few exceptions. Except for Mexico and Poland, the trend was significant for all other countries although it was in opposite direction (decreasing with increase in age in Russian Federation, Ukraine and Uruguay). The prevalence after adjusting for other cofactors was significantly higher for rural areas only in India and Thailand. The difference was not significant for Bangladesh, China and Philippines. For educational level, odds ratios were computed taking highest level of education (completed college) as the reference. Most odds ratios were significant with the largest difference being four fold in Bangladesh and Thailand. The trend was significant in Bangladesh, Egypt, India, Philippines and Thailand demonstrating decreasing prevalence of tobacco use with increasing level of education. The trend was in the opposite direction for Turkey and not significant for the rest of the countries.

**Table 3:** Predictors of current tobacco use among adults age 15 years and above in 13 low-and-middle income countries using logistic regression analysis, Global Adult Tobacco Survey, 2008–2010.

Socio-demographic characteristics	Bangladesh		China		Egypt		India		Mexico		Philippines		Poland	
	OR	95% CI	OR	95% CI	OR	95% CI	OR	95% CI	OR	95% CI	OR	95% CI	OR	95% CI
**Gender**														
Male	6.79	(5.9, 7.8)***	82.19	(63.7, 106.0)***	162.2	(110.9, 237.3)***	6.08	(5.6, 6.6)***	4.93	(4.3, 5.7)***	16.93	(14.4, 19.9)***	2.37	(2.1, 2.7)***
Female (RC)	1.0		1.0		1.0		1.0		1.0		1.0		1.0	

Age‡	p<.001		p<.001		p<.001		p<.001		p = .231		p<.001		p = .259	
15–24	1.0		1.0		1.0		1.0		1.0		1.0		1.0	
25–34	3.06	(2.5, 3.8)***	2.58	(1.9, 3.5)***	2.99	(2.5, 3.6)***	2.51	(2.3, 2.8)***	1.21	(1.0, 1.5)*	2.42	(1.9, 3.0)***	2.38	(1.8, 3.1)***
35–44	6.81	(5.4, 8.6)***	4.18	(3.1, 5.6)***	3.62	(3.0, 4.4)***	3.54	(3.2, 3.9)***	1.08	(0.8, 1.4)	2.56	(2.1, 3.2)***	2.69	(2.1, 3.5)***
45–54	12.8	(9.8, 16.7)***	5.68	(4.4, 7.4)***	4.34	(3.5, 5.4)***	4.31	(3.8, 4.9)***	1.31	(1.0, 1.7)*	3.05	(2.3, 4.0)***	3.51	(2.7, 4.6)***
55–64	19.09	(14.1, 25.9)***	4.54	(3.3, 6.2)***	3.36	(2.7, 4.2)***	4.88	(4.2, 5.6)***	0.99	(0.8, 1.3)	4	(3.0, 5.4)***	2.32	(1.8, 3.0)***
65+	17.99	(12.8, 25.3)***	2.66	(1.9, 3.8)***	2.38	(1.8, 3.1)***	4.68	(4.1, 5.4)***	0.62	(0.5, 0.8)**	4.45	(3.0, 6.6)***	0.62	(0.4, 0.9)**
**Place of residence**														
Urban (RC)	1.0		1.0		1.0		1.0		1.0		1.0		1.0	
Rural	0.94	(0.8, 1.1)	1.17	(0.9, 1.5)	0.83	(0.7, 0.9)**	1.24	(1.1, 1.4)***	0.64	(0.5, 0.8)***	1.02	(0.9, 1.2)	0.7	(0.6, 0.8)***

Education‡	p<.001		p = .404		p<.001		p<.001		p = .491		p<.001		p<.001	
No formal education/Less than primary	4.02	(2.6, 6.2)***	2.22	(1.4, 3.6)**	3.44	(2.8, 4.2)***	3.03	(2.6, 3.5)***	1.26	(0.9, 1.8)	3.38	(2.5, 4.5)***	0.88	(0.4, 2.1)
Completed primary/Less than secondary	2.29	(1.5, 3.5)***	1.65	(1.1, 2.4)*	2.13	(1.7, 2.7)***	1.96	(1.7, 2.3)***	1.25	(0.9, 1.7)	2.24	(1.7, 3.0)***	1.52	(1.2, 2.0)**
Completed secondary/high school	1.03	(0.7, 1.6)	1.74	(1.3, 2.4)***	1.68	(1.4, 2.0)***	1.19	(1.0, 1.4)*	1.17	(0.9, 1.6)	1.65	(1.3, 2.0)***	1.7	(1.4, 2.1)***
Completed college/university or above (RC)	1.0		1.0		1.0		1.0		1.0		1.0		1.0	

Wealth Index‡	p<.001		p = .878		p = .774		p<.001		p<.001		p<.001		p = .463	
Lowest	2.23	(1.7, 2.9)***	0.95	(0.7, 1.3)	1.2	(1.0, 1.5)	2.96	(2.6, 3.4)***	0.62	(0.5, 0.8)**	2.09	(1.6, 2.8)***	1.39	(1.1, 1.8)**
Low	1.69	(1.3, 2.2)***	1.28	(1.0, 1.7)	1.21	(1.0, 1.5)	2.27	(2.0, 2.6)***	0.66	(0.5, 0.9)**	2.2	(1.7, 2.9)***	1.32	(1.1, 1.6)*
Middle	1.44	(1.1, 1.8)**	1.17	(0.9, 1.4)	1.57	(1.3, 1.9)***	1.88	(1.7, 2.1)***	0.82	(0.7, 1.0)	1.88	(1.5, 2.4)***	1.3	(1.1, 1.6)*
High	1.4	(1.1, 1.8)**	1.35	(1.0, 1.8)*	1.32	(1.1, 1.6)**	1.46	(1.3, 1.6)***	0.95	(0.8, 1.1)	1.59	(1.2, 2.0)***	1.12	(0.9, 1.4)
Highest (RC)	1.0		1.0		1.0		1.0		1.0		1.0		1.0	

Knowledge on effects of smoking‡	p = .347		p<.001		p = .216		p<.001		p = .336		p<.001		p<.001	
Little knowledge	1.15	(0.9, 1.4)	1.52	(1.2, 1.9)***	1.15	(0.9, 1.5)	1.26	(1.2, 1.4)***	0.84	(0.7, 1.0)	1.76	(1.5, 2.1)***	2.57	(2.2, 3.0)***
Some knowledge	1.16	(0.9, 1.5)	1.01	(0.7, 1.4)	1.28	(1.0, 1.7)	1	(0.9, 1.1)	1.16	(1.0, 1.4)	1.21	(1.0, 1.5)	1.39	(1.2, 1.6)***
Good knowledge (RC)	1.0		1.0		1.0		1.0		1.0		1.0		1.0	

Note: OR-Odds Ratio; CI-Confidence Interval; RC-Reference Category;

*** p<0.001,

** p<0.01,

* p<0.05;

‡ p-values shown for test of linear trend.

**Table 4:** Predictors of current tobacco use among adults age 15 years and above in 13 low-and-middle income countries using logistic regression analysis, Global Adult Tobacco Survey, 2008–2010.

Socio-demographic characteristics	Russian Federation		Thailand		Turkey		Ukraine		Uruguay		Viet Nam	
	OR	95% CI	OR	95% CI	OR	95% CI	OR	95% CI	OR	95% CI	OR	95% CI
**Gender**												
Male	8.3	(7.1, 9.7)***	29.02	(25.1, 33.5)***	7.99	(6.9, 9.3)***	13.18	(11.1, 15.6)***	2.08	(1.8, 2.5)***	87.33	(68.3, 111.6)***
Female (RC)	1.0		1.0		1.0		1.0		1.0		1.0	

Age‡	p<.001		p<.001		p = .052		p<.001		p = .004		p<.001	
15–24	1.0		1.0		1.0		1.0		1.0		1.0	
25–34	2.06	(1.6, 2.7)***	2.18	(1.7, 2.8)***	2.87	(2.3, 3.6)***	2.71	(2.0, 3.7)***	2.08	(1.5, 2.9)***	3.81	(2.9, 5.0)***
35–44	2.05	(1.6, 2.7)***	2.57	(2.0, 3.2)***	3.05	(2.5, 3.8)***	2.08	(1.6, 2.8)***	1.36	(1.0, 1.9)	8.26	(6.3, 10.9)***
45–54	1.43	(1.1, 1.8)**	2.37	(1.8, 3.0)***	2.89	(2.3, 3.7)***	1.5	(1.1, 2.0)**	2.29	(1.6, 3.3)***	10.91	(8.1, 14.7)***
55–64	0.97	(0.7, 1.3)	3.86	(2.9, 5.2)***	2.11	(1.6, 2.8)***	0.71	(0.5, 1.0)*	1.5	(1.1, 2.1)*	13.18	(9.1, 19.2)***
65+	0.4	(0.3, 0.5)***	9.41	(6.9, 12.9)***	0.73	(0.5, 1.0)	0.29	(0.2, 0.4)***	0.3	(0.2, 0.5)***	12.76	(8.1, 20.1)***
**Place of residence**												
Urban (RC)	1.0		1.0		1.0		1.0		1.0		1.0	
Rural	0.67	(0.6, 0.8)***	1.18	(1.0, 1.3)**	0.78	(0.7, 0.9)**	0.56	(0.5, 0.7)***	0.6	(0.5, 0.8)***	0.8	(0.7, 1.0)*

Education‡	p = .421		p<.001		p<.001		p = .965		p = .764		p<.001	
No formal education/Less than primary	0.99	(0.4, 2.3)	4.21	(3.1, 5.7)***	0.96	(0.7, 1.4)	1.82	(0.2, 20.6)	1.51	(1.0, 2.3)	2.18	(1.5, 3.1)***
Completed primary/Less than secondary	0.91	(0.6, 1.3)	2.22	(1.7, 3.0)***	1.32	(1.0, 1.7)*	1.33	(0.9, 2.1)	1.18	(0.8, 1.7)	1.51	(1.1, 2.1)**
Completed secondary/high school	1.33	(1.1, 1.6)**	1.92	(1.5, 2.5)***	1.86	(1.4, 2.5)***	1.6	(1.3, 2.0)***	1.08	(0.7, 1.6)	1.21	(0.9, 1.6)
Completed college/university or above (RC)	1.0		1.0		1.0		1.0		1.0		1.0	

Wealth Index‡	p = .733		p<.001		p = .057		p = .001		p<.001		p<.001	
Lowest	1.14	(0.9, 1.5)	3.35	(2.6, 4.3)***	1.29	(1.0, 1.6)*	1.71	(1.2, 2.4)***	2.91	(2.0, 4.2)***	2.5	(1.8, 3.6)***
Low	0.83	(0.7, 1.0)	2.83	(2.2, 3.6)***	1.24	(1.0, 1.6)	1.57	(1.2, 2.1)**	1.61	(1.2, 2.2)**	1.92	(1.4, 2.6)***
Middle	0.97	(0.8, 1.2)	2.21	(1.7, 2.8)***	1.32	(1.1, 1.6)*	1.49	(1.2, 1.9)**	1.61	(1.2, 2.1)**	1.67	(1.3, 2.2)***
High	0.87	(0.7, 1.1)	1.65	(1.3, 2.0)***	1.12	(0.9, 1.4)	1.34	(1.0, 1.7)*	1.14	(0.8, 1.5)	1.34	(1.0, 1.8)*
Highest (RC)	1.0		1.0		1.0		1.0		1.0		1.0	

Knowledge on effects of smoking‡	p<.001		p = .059		p = .621		p<.001		p = .420		p<.001	
Little knowledge	2.78	(2.3, 3.3)***	1.2	(1.0, 1.5)	1.14	(0.9, 1.5)	1.93	(1.6, 2.4)***	1.71	(1.2, 2.4)**	1.71	(1.3, 2.2)***
Some knowledge	1.65	(1.3, 2.0)***	1.08	(0.9, 1.3)	1.11	(0.9, 1.3)	1.33	(1.0, 1.8)	0.84	(0.7, 1.1)	1.1	(0.9, 1.4)
Good knowledge (RC)	1.0		1.0		1.0		1.0		1.0		1.0	

Note: OR-Odds Ratio; CI-Confidence Interval; RC-Reference Category;

*** p<0.001,

** p<0.01,

* p<0.05;

‡ p-values shown for test of linear trend.

For wealth index, odds ratios were computed taking the highest wealth category as reference. Most of odds ratios were significant with largest effect observed in Thailand. The trend (decreasing odds of tobacco use with increasing wealth) was significant for Bangladesh, India, Philippines, Thailand, Turkey, Ukraine, Uruguay and Viet Nam. The trend was opposite in Mexico and not significant in other countries. Knowledge level was also affected tobacco use, though to a lesser extent. An inverse relationship was observed for level of knowledge and tobacco use; as level of knowledge increased, the odds of tobacco use decreased and was significant for China, India, Philippines, Poland, Russian federation, Thailand, Ukraine and Viet Nam. The trend was not significant in remaining countries.

## Discussion

This report provides information about 13 countries where 2.64 billion adults aged 15 years and above live, which constitutes more than half of the world's adult population (5.15 billion) in 2010 [29]. The findings from this report indicate that across these 13 countries over three-quarters of a billion (768.5 millions) are current tobacco users. Moreover, the findings provide evidence that social determinants are associated with tobacco use behavior. Most reports on tobacco use [4] are confined to smoking as it is the only form of tobacco use in most of the countries. Among these 13 countries however, smokeless tobacco use is the dominant form of tobacco use behavior in at least two countries (Bangladesh and India), making it inadvisable to leave out smokeless tobacco use while discussing tobacco use behavior. For the sake of uniformity, we decided to combine both smoking and smokeless tobacco use and termed it as ‘tobacco use’ for all countries. Questions about smokeless tobacco use were asked in all countries except Turkey. The findings show that in addition to Bangladesh and India, smokeless tobacco use was important for Thailand, Philippines and Viet Nam as well.

Our study reveal that the prevalence of current tobacco use, particularly smoking is quite lower among women compared with men in most countries and sex is a very strong determinant of tobacco use status. Stratified analysis of tobacco use by sex (not shown in tables) clearly indicated that the present findings apply to women as well as men. Our study also reveals that the prevalence of tobacco use is generally higher among urban, less educated and low economic groups and people with less knowledge about effects of smoking. Detailed questions about the health effects of smokeless tobacco use were asked only in Bangladesh. The level of knowledge (calculated similar to smoking) based on three specific diseases (stroke, heart attack, and cancer of mouth) that are caused by smokeless tobacco use showed that the smokeless tobacco use is higher among individuals with lower level of knowledge (little knowledge (30%), some knowledge (34.3%) and good knowledge (26.5%)).

An important finding in this study is high prevalence of tobacco use in the middle ages (45 to 64). The health effects of tobacco use start becoming apparent in these age groups in a major way [30]. Therefore, targeting cessation in these age groups would be extremely important as a component of overall policy initiatives for reducing tobacco use prevalence [31]. This will be crucial in reducing morbidity and mortality caused by tobacco use in the immediate future [32]. In general, social determinants associated with inequality such as education and wealth were correlated with increased tobacco use. However, some exceptions were seen. In a few countries increased wealth and education were not associated with decreased tobacco use, with Mexico actually having lower tobacco use in the poor, and with the lowest rates of tobacco use in China present in the poorest and wealthiest. Future research to understand the determinants of these patterns is warranted.

In this paper, we study social determinants as predictors of tobacco use, but in the long term tobacco use itself causes social inequalities [33]. In disadvantaged sections of society, expenditure on tobacco use often replaces expenditure on other essential items and services for the family. In the long term, these families suffer serious morbidity and mortality due to tobacco use which accentuates determinants further [34]. Intra-country differences in tobacco use influence the overall burden of disease and death and substantially contribute to overall between -country differences in other parameters of public health [35]. Monitoring of tobacco epidemic will be necessary to increase the effectiveness of existing public health strategies and for development of tailored interventions [36], particularly targeting young people and women [30] to stop using tobacco use and discourage initiation to reduce tobacco-related disparities.

At least two health parameters included in Millennium Development Goals (MDGs) are strongly related to tobacco use: deaths by tuberculosis and maternal and child health issues [37]. Currently about a billion adults use tobacco every day and about 15,000 die from tobacco-related diseases every day [38]. Smoking causes half of all male deaths among tuberculosis patients in India [39], [40]. Smoking by pregnant women is well established as a causative factor for low birth weight, still birth and other adverse reproductive outcomes. Recent evidence establishes that a non-cigarette form of tobacco use also causes adverse reproductive outcomes [41], especially smokeless tobacco use in India [42], [43]. In addition, there is a strong indication that exposure to secondhand smoke increases the risk of still birth [44]. Tobacco use accounts for one in six of all deaths resulting from Non-Communicable Diseases (NCDs) [45]. Socioeconomic impacts of NCDs are affecting the progress towards UN MDGs [45]. It is therefore clear that a high level of tobacco use especially among disadvantaged groups in these 13 countries is an important hindrance to the attainment of the MDGs.

The findings in this report are subject to a few limitations. The prevalence results are based on self-reports without bioassay validation. Study design allowed for the investigation of only a limited number of socio-demographic variables. It is important to note that former tobacco users were excluded from the logistic regression. The proportion of former users was different in different countries and their distribution by socio-demographic variables used in the analysis might be different. This might affect some comparisons. The information on frequency and length of smoking, though available in GATS data, was not considered in the present study. In some countries the household possession items considered/used in the analysis are based on the items available in the country data and these items may not be a true representation of wealth across all countries.

Despite these limitations, the current study provides evidence of the importance of social determinants on tobacco use. Findings indicate that social determinants and their role should be given high priority when addressing the issue of tobacco use.

## Supporting Information

List S1 **GATS Collaborating Group.**
(DOCX)
