# Supplementary material for: Social Determinants of Health and Tobacco Use in Thirteen Low and Middle Income Countries: Evidence from Global Adult Tobacco Survey
Source: PLoS One. 2012 Mar 16;7(3):e33466. doi: 10.1371/journal.pone.0033466 (PMC3306395; doi:10.1371/journal.pone.0033466)
Supplement: List S1 — GATS Collaborating Group. (DOCX) [file pone.0033466.s001.docx]

**List S1 GATS Collaborating Group**

| National Agencies and Collaborators | | |
| --- | --- | --- |
|  |  |  |
| **Country Name** | **Agencies** | **Collaborators** |
|  |  |  |
| **Bangladesh** | Ministry of Health and Family Welfare (MoHFW), National Institute of Preventive & Social Medicine (NIPSOM), Bangladesh Bureau of Statistics (BBS) National Institute of Population, Research & Training (NIPORT) | Md. Amirul Hasan (NIPSOM) |
| **Brazil** | Ministry of Health-Secretariat of Health Surveillance (SVS), Brazilian Institute of Geography & Statistics (IBGE), National Cancer Institute (INCA), The National Health Surveillance Agency (ANVISA) | Deborah Carvalho Malta (MoH-SVS), Eduardo Pereira Nunes, Marcia Quintslr, Cimar Azeredo (IBGE), Liz Maria de Almeida (INCA), Humberto Martins (ANVISA) |
| **China** | Ministry of Health (MoH), Chinese Centers for Disease Control (China CDC) | Yang Gonghuan, Yang Yan, Xiao Lin, Li Qiang (China CDC) |
| **Egypt** | Ministry of Health (MoH) Central Agency for Public Mobilization & Statistics (CAPMAS) | Sahar Latif Labib (MoH), Awatef Hussein (CAPMAS) |
| **India** | Ministry of Health & Family Welfare (MoHFW) ― Government of India, International Institute for Population Sciences (IIPS) | Anuradha Vemuri, Jagdish Kaur (MoHFW), F. Ram, Sulabha Parasuraman (IIPS) |
| **Mexico** | Ministry of Health (MoH) National Institute of Public Health (INSP) | Mauricio Hernandez Avila (MoH), Luz Miriam Reynales-Shigematsu (INSP) |
| **Philippines** | Department of Health (DoH), National Statistics Office (NSO) | Agnes Segarra (DoH), Glenn Barcenas, Benedicta Yabut (NSO) |
| **Poland** | Ministry of Health (MoH), Maria Skłodowska-Curie Cancer Center Institute of Oncology, Medical University of Warsaw, Pentor Research International | Tadeusz Parchimowicz (MoH), Witold Zatonski, Krzysztof Przewozniak (CCI), Filip Raciborski (WMU), Krzysztof Siekierski (Pentor) |
| **Russian Federation** | Ministry of Health & Social Development (MoHSD), Federal State Statistics of Russia (RosStat), Pulmonary Research Institute (PRI) | Maria Shevireva, Natalya Kostenko, (MoHSD), Vadim Nesterov, Tamara Chernisheva, Tatiana Konik (RosStat), Galina Sakharova (PRI) |
| **Thailand** | Ministry of Public Health (MoPH), National Statistical Office (NSO),  Tobacco Control Research & Knowledge Management Center (TRC) at Mahidol University | Sarunya Benjakul (MoPH), Lakkhana Termsirikulchai, Mondha Kengganpanich (TRC), Areerat Lohtongmongkol, Hataichanok Puckcharern, Chitrlada Touchchai (NSO) |
| **Turkey** | Ministry of Health (MoH), Turkish Statistical Institute (TurkStat), Hacetteppe University (HU) | Hüseyin İlter (MoH), Enver Tasti, Ramazan Celikkaya, Guzin Erdogan (Turkstat), Nazmi Bilir, Hilal Özcebe (HU) |
| **Ukraine** | Ministry of Health (MoH), Kiev International Institute of Sociology (KIIS), School of Public Health, National University of Kyiv-Mohyla Academy (SPH) | Alla Grygorenko, Konstantin Krasovsky (MoH), Natalia Kharchenko, Volodymyr Paniotto (KIIS), Tatiana Andreeva (SPH) |
| **Uruguay** | Ministry of Health (MoH), National Statistics Institute (INE) | Winston Abascal, Ana Lorenzo (MoH), Franco González Mora (INE) |
| **Viet Nam** | Ministry of Health (MoH), Vietnam Standing Office on Smoking and Health (VINACOSH), General Statistics Office (GSO), Hanoi Medical University (HMU) | Phan Thi Hai (MoH), Nguyen The Quan (GSO), Hoang Van Minh (HMU), Kim Bao Giang (HMU) |

International Agencies and Collaborators

**World Health Organization (WHO) ─ Tobacco Free Initiative**

Headquarters: Douglas Bettcher, Lubna Bhatti, Edouard Tursan d’Espaignet, Sameer Pujari, Ayda Yurekli

AFRO: A.E. Ogwell Ouma, Nivo Ramanandraibe

*AMRO-PAHO:* Adriana Blanco, Roberta de Betania Caixeta

*Country Offices: Brazil:* Enrique Gil; *Mexico:* Carlos Gamez; *Uruguay:* Julio Vignolo

EMRO: Fatimah El Awa, Heba Fouad

*Country Office - Egypt:* Randa Abou El Naga

EURO: Kristina Mauer-Stender, Rula Khoury

*Country Offices: Poland:* Anna Koziel; *Russian Federation:* Luigi Migliorini, Oleg Storozhenko; *Turkey:* Toker Ergüder;

*Ukraine:* Nataliya Korol

SEARO: Dhirendra N. Sinha

*Country Offices: Bangladesh*: Sohel Choudhury, M. Mostafa Zaman; *India:* Vineet Munish Gill; *Thailand:* Chai Kritiyapichatkul

WPRO: Susan Mercado, James Rarick

*Country Offices: China:* Sarah England; *Philippines*: Marina Miguel-Baquilod; *Vietnam*: Pham Thi Quynh Nga, Pham Huyen Khanh

**U.S. Centers for Disease Control and Prevention (CDC)**Global Tobacco Control Branch, Office on Smoking and Health (OSH)

Linda Andes, Samira Asma (Branch Chief), Glenda Blutcher-Nelson, Felicita David, Peter Edwards, Thomas R. Frieden (CDC Director), Jason Hsia, Deliana Kostova, Ronney Lindsey, Charity “Nikki” Mayes, Timothy McAfee (OSH Director), Sara Mirza, Jeremy Morton, Krishna Mohan Palipudi, Terry Pechacek, Edward Rainey, Dana Shelton, Yang “Sophia” Song, Raydel Valdés Salgado, Brian Taitt, Luhua Zhao

**CDC Foundation**

William Parra, Brandon Talley, Connie Granoff, Michael Green

**Johns Hopkins Bloomberg School of Public Health**

Joanna Cohen, Rajeev Cherukupalli

**RTI International**

Steve Litavecz

**Experts & Scientific Advisors**

*Questionnaire Review Committee*

Benjamin Apelberg, Jeremy Morton, Marina Miguel-Baquilod, Ron Borland, Gary Giovino, Prakash C. Gupta, Ahmed Mandil.

*Sample Review Committee*

Michael Bowling, William Kalsbeek, Krishna Mohan Palipudi, T. K. Roy

*Scientific Advisors*

Sonia Angell , Neeraj Bhalla, Frank Chaloupka, Prabhat Jha, Judith Mackay, Sir Richard Peto, Jonathan Samet, Gajalakshmi Vendhan, Witold Zatonski

**Bloomberg Philanthropies – Bloomberg Initiative to Reduce Tobacco Use**

Kelly Henning, Jennifer Ellis
